# Obstructive sleep apnea severity and pathophysiological traits in overlap syndrome: Insights from the SNOOzzzE cohort

**DOI:** 10.14814/phy2.70438

**Published:** 2025-07-03

**Authors:** Janna Raphelson, Ana Sanchez‐Azofra, Jeremy E. Orr, Gabriela Parra, Lana McGinnis, Alexis Salinas, Steven Luu, Scott A. Sands, Ali Azarbarzin, Robert L. Owens, Jose M. Marin, Atul Malhotra, Christopher N. Schmickl

**Affiliations:** ^1^ Division of Pulmonary, Critical Care, Sleep Medicine and Physiology University of California, San Diego (UCSD) San Diego California USA; ^2^ Division of Pulmonary and Sleep Medicine Hospital Universitario de la Princesa, Universidad Autónoma de Madrid Madrid Spain; ^3^ Apnimed Inc. Cambridge Massachusetts USA; ^4^ Division of Sleep and Circadian Disorders Brigham and Women's Hospital and Harvard Medical School Boston Massachusetts USA; ^5^ Hospital Universitario Miguel Servet, IIS Aragón‐CIBERES and Department of Medicine University of Zaragoza Zaragoza Spain

**Keywords:** chronic obstructive pulmonary disease, endotypes, obstructive sleep apnea

## Abstract

The overlap syndrome (OVS), defined as coexisting chronic obstructive pulmonary disease (COPD) and obstructive sleep apnea (OSA), is linked to worse outcomes than either condition alone. Patients with COPD and OSA may have fewer obstructive events, but underlying mechanisms remain unclear. Using a large clinical cohort, we tested the hypothesis that OSA severity and pathophysiological traits differ in OVS versus OSA‐alone. Data from the SNOOzzzE cohort (3319 adults with in‐laboratory polysomnography 2017–2019) were used. OVS patients were identified through chart review and matched to OSA‐only patients (3:1) by age, sex, and body mass index. OSA severity was assessed using apnea hypopnea index (AHI), hypoxic burden (HB), and T90 (%time with SpO_2_ < 90%), while OSA traits were quantified from polysomnographic signals via validated algorithms. Mixed model analysis quantified group differences before and after adjustment for covariate differences (Black race, smoking) accounting for matching as a random effect. In our diverse cohort (103 OVS vs. 309 OSA‐only; 38% women, 44% non‐White, 17% Hispanic), OVS patients tended to have a lower AHI and HB (approximately −10%, *p* < 0.1), but significantly higher T90 (~50%, *p* = 0.003). OVS patients had less upper airway collapsibility, lower arousal threshold, lower ventilatory response to arousal (*p* < 0.05) and tended to have higher upper airway dilator muscle compensation (*p* = 0.09). In adjusted analyses, effect estimates were similar, but significance was attenuated. Hyperinflation and air trapping were inversely associated with AHI/HB. OSA severity and mechanisms differ in OVS versus OSA‐only. Future research should seek to evaluate these differences for their prognostic ability.

## 1 INTRODUCTION

The “overlap syndrome” (OVS), defined as coexisting chronic obstructive pulmonary disease (COPD) and obstructive sleep apnea (OSA), is associated with worse outcomes (e.g., quality of life (van Zeller et al., 2023), cardiovascular fitness (de Carvalho Junior et al., 2020), morbidity (Sanchez‐Azofra et al., 2023; Sun et al., 2019; Wang et al., 2020), and mortality (Jaoude & El‐Solh, 2019)) compared to patients with COPD or OSA alone. Previous studies have suggested that OSA may be characterized by fewer respiratory events in patients with OVS compared to those with OSA alone (i.e., lower apnea‐hypopnea index [AHI]) (Hashiguchi et al., 2023) in OVS than OSA alone, but have provided limited insights regarding the pathophysiological reasons for this finding.

OSA is a highly prevalent disease (Benjafield et al., 2019) characterized by repetitive narrowing of the upper airway during sleep resulting in cessation of airflow with preserved or increased respiratory effort (Sunwoo & Malhotra, 2024). These events can lead to sympathetic activation and sleep fragmentation, resulting in increased risk of cardiometabolic and neurocognitive impairments (Jordan et al., 2014). However, OSA symptoms and sequelae vary across patients, in part likely due to differences in underlying mechanisms. While all OSA patients have some anatomical predisposition to upper airway collapse, in the majority of patients nonanatomical traits are critical for OSA pathogenesis (Eckert et al., 2013): For example, unstable respiratory control (“high loop gain”) or large ventilatory response to arousals can lead to intermittently decreased input to pharyngeal dilator muscles and thus collapse. Moreover, while normal pharyngeal dilator muscles rapidly respond to increasing respiratory stimuli (e.g., increase in CO_2_) following airway narrowing, in a subgroup of OSA patients the dilator muscles do not respond vigorously enough to restore upper airway patency and thus maintain sleep. Similarly, OSA patients with a low arousal threshold may wake up before upper airway dilators can fully engage. Importantly, there is increasing evidence that the underlying mechanisms (or “endotypic traits”) driving OSA in individual patients may predict OSA sequelae and response to therapy (Edwards et al., 2019; Malhotra et al., 2020; Schmickl et al., 2024), which may in part explain why to date trials regarding cardiovascular outcomes with OSA treatment with positive pressure in general OSA patients have been disappointing despite clear anecdotal and clinical successes (Barbé et al., 2012; Phillips et al., 2013; Traaen et al., 2021).

Understanding the underlying mechanisms of OSA in patients with COPD may help explain their poor cardiac outcomes, and ultimately facilitate a personalized treatment approach. Thus, leveraging a recent technique to measure pathophysiological traits underlying OSA from routine sleep studies in a relatively large clinical cohort, we tested the hypothesis that OSA severity and traits differ in OVS versus OSA‐alone, and explored whether pulmonary function test (PFT) abnormalities in OVS are associated with OSA severity and traits.

## 2 METHODS

### 2.1 Study population

The San Diego Multi‐Outcome OSA Endophenotype (SNOOzzzE) cohort includes 3319 consecutive adults who underwent a clinical in‐laboratory polysomnography at the UCSD sleep clinic between 1/2017 and 12/2019. This retrospective cohort research was reviewed and approved by the UCSD Institutional Review Board (#182136), and was granted a waiver of informed consent. For this analysis we included patients with OSA (i.e., apnea hypopnea index, AHI >5/h), who's in‐laboratory polysomnography allowed for advanced signal analyses (see below). We excluded patients who had predominantly central sleep apnea.

### 2.2 Matching

The goal was to match patients with OVS (COPD+OSA) with similar patients who have OSA only. Thus, past medical history and active problem lists in the notes from sleep providers just prior and after the polysomnography were queried for terms indicating COPD (“copd”, “emphysema”, “chronic obstructive pulmonary”, and “obstructive lung”). COPD status at the time of the polysomnography was then verified via a manual chart review by trained pulmonologists (ASA and JR) taking into account any available notes and/or pulmonary function tests when present. Patient's whose COPD status was verified, were then matched in a 1:3 ratio with participants from the remaining cohort who had the same sex, similar age (±5 years), and body mass index (±3 kg/m^2^). The matched “OSA only” participants were then manually reviewed to verify absence of COPD; patients who were found to have COPD on manual review were included in the matching procedure as part of an iterative process.

### 2.3 Measurements of OSA severity

OSA severity was primarily quantified using the apnea hypopnea index (AHI3A), with hypopneas defined as a reduction in flow by ≥30% for ≥10 s associated with an arousal or oxygen desaturation by ≥3%. In addition, we quantified hypoxemia using the sleep‐apnea specific hypoxic burden (HB), measured as the average area under the event‐related desaturation curve (Azarbarzin et al., 2019), and as the percentage of total sleep time (TST) spent with an oxygen saturation below 90% (T90). Complementary metrics included the AHI3A during rapid eye movement (REM) and non‐REM (NREM) sleep, the AHI4 based on hypopneas with oxygen desaturations by ≥4%, as well as mean and nadir oxygen saturation (SpO_2_) during sleep.

### 2.4 Measurements of OSA traits

Pathophysiological traits underlying OSA were estimated from raw signals obtained during NREM‐sleep portions of the in‐laboratory polysomnography using published techniques (Azarbarzin et al., 2019; Heinzer et al., 2006). To assess upper airway collapsibility we quantified Vpassive, which estimates the flow as a percentage of eupneic ventilation (%Veupnea, %VE) at eupneic drive (lower Vpassive denotes worse collapsibility). To assess responsiveness of upper airway dilator muscles, we quantified Vcomp which reflects the change in flow from passive to active conditions (higher Vcomp denotes better upper airway dilator muscle response). Ventilatory instability (“loop gain”) was estimated as the magnitude of the ventilatory drive response to a prior reduction in ventilation in the time frame of 1 min (range 0 to infinity, higher levels of loop gain denote more instability). The arousal threshold was estimated as the level of respiratory drive causing arousals (lower values denoting easier arousability). Furthermore, the ventilatory response to arousal was estimated as the ventilatory overshoot (%VE) in response to arousals (greater overshoots may worsen sleep apnea) (Edwards et al., 2013; Jordan et al., 2004). Vpassive (Vpassive^T^ = 100‐100*sqrt[1‐Vpassive/100]) and Arousal Threshold (Arousal Threshold^T^ = 100 + 100*sqrt[ArTH/100–1]) were transformed for normality as in prior studies with transformed values used throughout (O'Driscoll et al., 2019; Sands et al., 2018) In addition, for descriptive analyses, traits were also dichotomized into high versus low based on previously published cut‐offs: low Vpassive^T^ (<77.6%VE, “High Collapsibility”) (O'Driscoll et al., 2019), low Vcomp (<0%VE, “Low Compensation”) (O'Driscoll et al., 2019), high Loop Gain 1 (>0.7, “Unstable Ventilatory Control”) (Terrill et al., 2015), and low Arousal Threshold^T^ (<145%VE) (O'Driscoll et al., 2019). Signal analysis was performed in MATLAB.

### 2.5 Pulmonary function tests

Clinical pulmonary function tests (PFT) were performed for each patient in our site's pulmonary function lab according to ATS standards. Tests were between 6‐ months to 4 years from PSG study date. If multiple PFTs were available for a patient the study that temporally closest to the studied PSG was referenced. Values from these tests including spirometry (forced expiratory volume in 1 s (FEV1) and forced vital capacity (FVC)), plethysmography (total lung capacity (TLC), residual volume (RV), functional residual capacity (FRC)), and diffusing capacity (DLCO) were obtained from the electronic medical record.

### 2.6 Statistical analysis

After matching OVS patients with OSA only patients, data were summarized as mean (standard deviation, SD) or *n* (%) using linear and logistic mixed effects models for univariable comparisons while including matching clusters as random effects (to account for the potential non‐independence of observations within each cluster).

Similarly, linear mixed effects models, accounting for matching clusters as random effects, were used to assess the fixed effect of COPD status on OSA severity and traits while adjusting for imbalances in race, ethnicity, and smoking status. Based on these analyses, means and 95%‐confidence intervals (CI) for OSA severity and traits in OVS versus OSA only patients were estimated by averaging across all levels of covariates for visualizations. Moreover, in OVS patients with complete pulmonary function test (PFT) data (*n* = 69), associations between PFT measures and OSA severity/traits were assessed as standardized coefficients using linear regression (bivariably and adjusted). As part of a sensitivity analysis, assessments were repeated in the subset of OVS patients (plus their matched counterparts) who had PFT‐proven COPD (diagnosis + FEV1/FVC <0.7) and did not use oxygen during the overnight polysomnography.

All analyses were performed using R (version 4.4.1, key packages: Matching, lmerTest, and ggeffects) (Team RC, 2019), with *p* values <0.05 considered statistically significant. Based on visual inspections, continuous variables were log‐ (adding 1 when values included 0) or square‐root transformed as needed to improve normality for inferential statistics.

## 3 RESULTS

**FIGURE 1:**
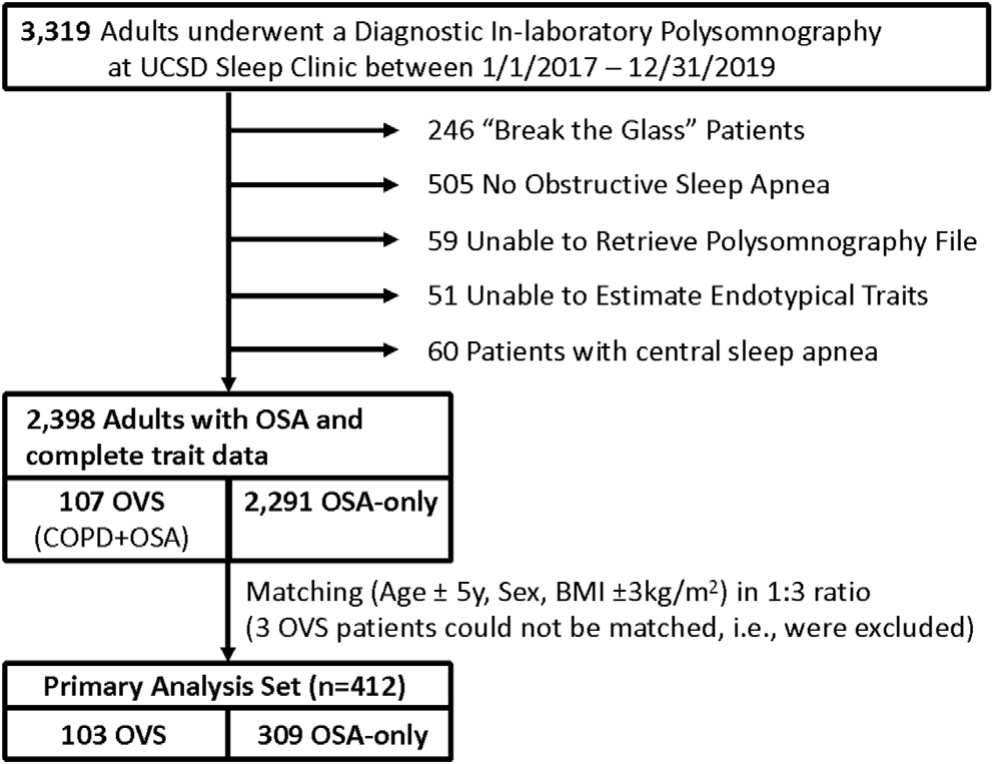
Study flowchart. “Break the glass” status denotes patients whose personal health information is deemed particularly sensitive and thus were excluded. OVS, overlap syndrome (OSA + COPD). “Break the glass” patients include some hospital employees, patients deemed to be at risk if their location became public at the treating team's discretion, and patient's who have requested additional security measures be added to their record.

We included 412 patients (103 OVS and 309 matched OSA only; Figure 1) in the primary analyses. Overall, the cohort was middle‐aged and relatively diverse, including 38% women, 44% non‐Whites, and 17% Hispanics/Latinos (Table 1). By design, OVS and OSA only patients were well matched with regard to age, sex, and BMI, but OVS patients were more likely to report African American race, active smoking, and less likely Hispanic/Latino ethnicity. Approximately one third of COPD patients had severe COPD based on a GOLD class of 3–4, and OVS patients reported slightly higher levels of daytime sleepiness based on the Epworth sleepiness score (*p* = 0.04).

**TABLE 1:** General characteristics. Some variables had to be transformed for normality, in which case descriptive statistics are shown both on the original and transformed scale, while inferential statistics (*p* values) were derived from transformed data only.

Characteristic	Overall	OVS	OSA only	*p* ^b^
	*N* = 412^a^	*N* = 103^a^	*N* = 309^a^	
Age, years	65 (9)	65 (9)	65 (9)	–
Female sex	156 (38%)	39 (38%)	117 (38%)	–
Body mass index, kg/m^2^	31 (7)	31 (7)	31 (7)	–
Race				
Asian	51 (12%)	13 (13%)	38 (12%)	0.01
Black or African American	28 (6.8%)	14 (14%)	14 (4.5%)	
White	231 (56%)	60 (58%)	171 (55%)	
Other or unknown	102 (25%)	16 (16%)	86 (28%)	
Hispanic/Latino ethnicity	72 (17%)	8 (7.8%)	64 (21%)	0.004
GOLD class				
Gold 1	16 (18%)	16 (18%)	–	
Gold 2	40 (45%)	40 (45%)	–	
Gold 3	29 (33%)	29 (33%)	–	
Gold 4	4 (4.5%)	4 (4.5%)	–	
Unknown	323	14	–	
FEV1, %predicted (*n* = 89)	61 (22)	61 (22)	–	
TLC, %predicted (*n* = 72)	94 (18)	94 (18)	–	
Current smoker	22 (5.3%)	16 (16%)	6 (1.9%)	<0.001
Epworth Sleepiness Score, ESS (*n* = 322)	8.3 (5.4)	9.3 (5.7)	8.0 (5.3)	
Sqrt(ESS) (*n* = 322)	2.67 (1.09)	2.90 (0.97)	2.60 (1.11)	0.04
Abnormal ESS, >10 (*n* = 322)	101 (31%)	27 (36%)	74 (30%)	0.28
General polysomnography results				
Oxygen used during overnight	8 (1.9%)	8 (7.8%)	0 (0%)	<0.001
Sleep efficiency, %	71 (17)	69 (18)	71 (16)	0.17
NREM Stage1, %	15 (14)	15 (16)	15 (13)	0.78
NREM Stage2, %	63 (15)	63 (17)	63 (15)	>0.9
NREM Stage3, %	11 (14)	10 (12)	11 (15)	0.43
REM sleep, %	11 (9)	12 (9)	11 (8)	0.43
Arousal index, total, h^−1^	48 (29)	47 (26)	48 (30)	0.56
Mean event duration, s	26 (6)	24 (6)	26 (6)	0.01
Mean pulse, min^−1^ (*n* = 411)	67 (12)	70 (13)	66 (11)	0.01
Max pulse, min^−1^ (*n* = 410)	87 (14)	90 (15)	86 (13)	0.02
OSA severity metrics				
Primary metrics				
AH3A, h^−1^	35 (24)	32 (23)	36 (24)	
Log(AHI3A), h^−1^	3.29 (0.77)	3.19 (0.75)	3.32 (0.77)	0.09
Hypoxic burden, %min/h	81 (87)	72 (86)	84 (87)	
Log(HB + 1), %min/h	3.93 (1.03)	3.81 (1.01)	3.97 (1.03)	0.12
T90, %	9 (17)	17 (26)	7 (12)	
Log(T90 + 1), %	1.45 (1.28)	1.79 (1.51)	1.34 (1.17)	0.001
Complementary metrics				
AHI3A_NREM_, h^−1^	34 (25)	30 (24)	35 (25)	
Sqrt(AHI3A_NREM_), h^−1^	3.20 (0.88)	3.08 (0.86)	3.24 (0.88)	0.07
AHI3A_REM_, h^−1^ (*n* = 318)	37 (25)	37 (27)	37 (24)	
Log(AHI3A_REM_), h^−1^ (*n* = 318)	5.61 (2.31)	5.60 (2.43)	5.61 (2.27)	0.85
AHI 4%, h^−1^	25 (23)	23 (22)	26 (23)	
Log(AHI4 + 1), h^−1^	2.80 (1.06)	2.69 (1.05)	2.84 (1.06)	0.17
SpO_2_ nadir, %	80 (9)	80 (9)	80 (10)	0.63
SpO_2_ mean, %	93.5 (2.3)	92.7 (3.0)	93.8 (2.0)	<0.001
OSA traits				
Vpassive^T^, %VE	64 (23)	68 (22)	63 (23)	0.045
High collapsibility (Vpassive^T^ < 77.6%)	296 (72%)	64 (62%)	232 (75%)	0.01
Vcomp, %VE	7 (19)	9 (16)	6 (20)	0.09
Low compensation (Vcomp<0%VE)	89 (22%)	17 (17%)	72 (23%)	0.15
Loop gain (dimensionless)	0.61 (0.18)	0.61 (0.20)	0.61 (0.17)	0.73
High loop gain (>0.7)	118 (29%)	32 (31%)	86 (28%)	0.51
Arousal threshold^T^, %VE	147 (28)	141 (27)	149 (28)	0.01
Low ArTH^T^ (<145%VE)	218 (53%)	63 (61%)	155 (50%)	0.053
Ventilatory response to arousal, %VE	40 (26)	33 (25)	42 (26)	0.002

*Note*: ^T^Transformed trait, see methods for details.

Abbreviations: %VE, ventilatory overshoot; AHI, apnea hypopnea index; ArTH, arousal threshold; NREM, non rapid eye movement sleep; OSA, obstructive sleep apnea; OVS, overlap syndrome; REM, rapid eye movement sleep; SpO_2_, oxygen saturation by pulse oximetry; TLC, total lung capacity.

^a^ Mean (SD); *n* (%).

^b^ Linear or logistic mixed effects for continuous and categorical characteristics, respectively.

### 3.1 Differences in OSA severity in OVS versus OSA only

**FIGURE 2:**
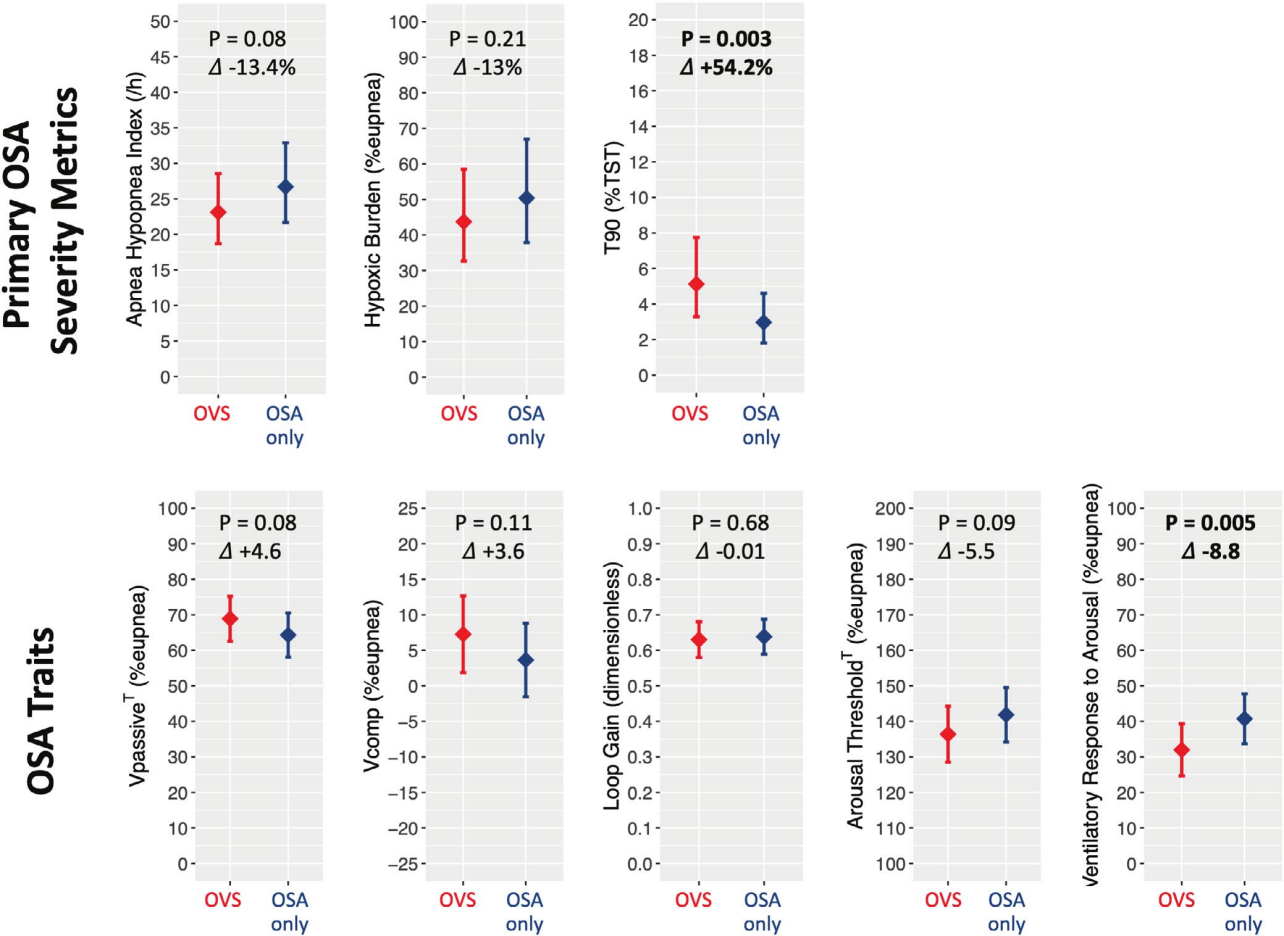
Estimated means and 95% confidence intervals for the primary OSA severity metrics (top panel) and OSA traits (bottom panel) in OVS versus OSA only Patients. Estimates for OVS versus OSA only are based on mixed effects models adjusting for race, ethnicity, and smoking status, and averaging across levels of covariates. OSA severity metrics were log‐transformed for analyses, with results being back‐transformed for visualization. Thus, for OSA metrics the 𝛥 reflects a percent difference, while for OSA traits the 𝛥 reflects the absolute difference in %eupnea.

Compared to OSA only patients, those with OVS tended to have a lower AHI3A (especially during NREM sleep) and HB (~10%, *p* < 0.1), but had a ~50% higher T90 (*p* ≥ 0.003) in univariable and adjusted analyses (Table 2, Figure 2). Of note, while sleep architecture was similar across groups, OVS patients had higher mean and maximum pulse rate possibly indicating higher cardiovascular tone.

**TABLE 2:** Results from univariable and multivariable linear mixed effects regression analyses. Results show the difference in OSA severity metrics and traits in OVS versus OSA only patients.

Outcome variable	Univariable models			Multivariable models (adjusted for race, ethnicity, and smoking)		
	*β* ^a^	(95% CI)	*p*	*β* ^a^	(95% CI)	*p*
Primary OSA severity metrics						
Log(AHI3A), h^−1^	−0.13	(−0.28 to 0.02)	0.09	−0.14	(−0.3 to 0.02)	0.08
Log(HB + 1), %min/h	−0.16	(−0.37 to 0.04)	0.12	−0.14	(−0.36 to 0.08)	0.21
Log(T90 + 1), %	0.45	(0.18 to 0.72)	0.001	0.43	(0.15 to 0.72)	0.003
OSA traits						
Vpassive^T^, %VE	4.88	(0.13 to 9.64)	0.045	4.58	(−0.43 to 9.58)	0.08
Vcomp, %VE	3.71	(−0.54 to 7.95)	0.09	3.63	(−0.78 to 8.04)	0.11
Loop gain (dimensionless)	−0.01	(−0.04 to 0.03)	0.73	−0.01	(−0.05 to 0.03)	0.68
Arousal threshold^T^, %VE	−7.62	(−13.68 to −1.56)	0.01	−5.47	(−11.8 to 0.85)	0.09
Ventilatory response to arousal, %VE	−9.03	(−14.78 to −3.29)	0.002	−8.75	(−14.78 to −2.72)	0.005

^a^ For log‐transformed variables the unstandardized beta reflects a difference on the log scale between OVS versus OSA only patients, which can be converted to a relative percent difference as follows: For example, for Log(AHI3A) the adjusted β of −0.14 reflects a (e^−0.14^‐1)*100 = −13% lower AHI in OVS versus OSA‐only; for all other variables the beta reflects the absolute difference between both groups on the native scale (e.g., absolute difference in %VE for Vpassive^T^).

### 3.2 Differences in OSA traits in OVS versus OSA only

In univariable analyses, OVS patients had significantly less upper airway collapsibility (i.e., higher Vpassive), a lower arousal threshold, a lower ventilatory response to arousal (*p* < 0.05), and tended to have a higher upper airway dilator muscle compensation (Vcomp, *p* = 0.09) than patients with OSA only (Table 2). When adjusting for covariates, effect estimates were similar, but significance was only maintained for the ventilatory response to arousal (Table 2, Figure 2).

### 3.3 PFT measures and OSA severity/traits in OVS patients

In bivariable analyses, higher levels of RV and FRC were associated with lower AHI and HB; in addition, higher TLC was associated with lower HB (Table 3). TLC was associated with lower T90, but this did not reach statistical significance. In fully adjusted analyses, results were similar, except that the relationship between TLC and HB became nonsignificant while a positive association between AHI and both FEV1 and FVC emerged (Table S1).

**TABLE 3:** Bivariable associations between PFT measures and OSA severity metrics/traits in OVS patients who had complete PFT data (*n* = 69). The standardized beta coefficients β_S_ reflect SD‐changes in the shown outcome variable for a 1‐standard deviation change of the PFT measure.

Outcome variable	FEV1 (%)	FVC (%)	RV (%)	FRC (%)	TLC (%)	RV/TLC (%)
	β_S_	β_S_	β_S_	β_S_	β_S_	β_S_
	(95% CI)	(95% CI)	(95% CI)	(95% CI)	(95% CI)	(95% CI)
	*p* Value	*p* Value	*p* Value	*p* Value	*p* Value	*p* Value
Primary OSA severity metrics						
Log(AHI3A), h^−1^	0.23	0.12	−0.31	−0.31	−0.14	−0.12
	(−0.01 to 0.47)	(−0.12 to 0.36)	(−0.54 to −0.07)	(−0.54 to −0.08)	(−0.38 to 0.11)	(−0.36 to 0.12)
	0.059	0.33	0.01	0.01	0.27	0.32
Log(HB + 1), %min/h	0.14	0.04	−0.35	−0.38	−0.24	−0.13
	(−0.10 to 0.38)	(−0.21 to 0.28)	(−0.58 to −0.13)	(−0.60 to −0.15)	(−0.48 to −0.003)	(−0.37 to 0.11)
	0.25	0.77	0.003	0.002	0.047	0.28
Log(T90 + 1), %	−0.18	−0.18	−0.09	−0.12	−0.21	0.06
	(−0.42 to 0.06)	(−0.42 to 0.06)	(−0.33 to 0.15)	(−0.37 to 0.12)	(−0.45 to 0.03)	(−0.19 to 0.3)
	0.15	0.14	0.46	0.31	0.08	0.65
OSA traits						
Vpassive^T^, %VE	0.01	−0.04	0.05	0.01	0.05	−0.11
	(−0.23 to 0.26)	(−0.28 to 0.21)	(−0.19 to 0.30)	(−0.23 to 0.26)	(−0.19 to 0.30)	(−0.35 to 0.13)
	0.93	0.78	0.66	0.92	0.66	0.37
Vcomp, %VE	−0.07	−0.13	−0.06	−0.12	−0.15	−0.04
	(−0.31 to 0.18)	(−0.37 to 0.11)	(−0.30 to 0.19)	(−0.36 to 0.13)	(−0.39 to 0.10)	(−0.29 to 0.2)
	0.60	0.28	0.63	0.35	0.23	0.72
Loop gain (dimensionless)	0.04	0.01	0.10	−0.04	0.06	−0.10
	(−0.20 to 0.29)	(−0.23 to 0.26)	(−0.15 to 0.34)	(−0.29 to 0.20)	(−0.19 to 0.30)	(−0.35 to 0.14)
	0.73	0.91	0.43	0.74	0.64	0.39
Arousal threshold^T^, %VE	0.01	−0.02	−0.03	−0.08	−0.09	0.01
	(−0.24 to 0.25)	(−0.26 to 0.23)	(−0.28 to 0.21)	(−0.33 to 0.16)	(−0.33 to 0.15)	(−0.24 to 0.25)
	0.95	0.89	0.80	0.50	0.46	0.94
Ventilatory response to arousal, %VE	0.04	0.06	−0.03	0.08	0.06	−0.01
	(−0.21 to 0.28)	(−0.18 to 0.31)	(−0.28 to 0.21)	(−0.16 to 0.33)	(−0.19 to 0.30)	(−0.26 to 0.23)
	0.77	0.61	0.78	0.50	0.66	0.93

### 3.4 Sensitivity analyses

When repeating the above analyses in the subset of PFT‐proven COPD patients who did not use oxygen during the overnight polysomnography (*n* = 64 OVS + 192 matched OSA only) the statistical significance of some of the findings was attenuated, but effect estimates were overall similar, suggesting robustness of the results (Tables S2 and S3).

## 4 DISCUSSION

The main finding of our study is that OVS patients tend to have milder OSA based on AHI and hypoxic burden (which captures primarily intermittent, OSA‐related hypoxia) than OSA patients without COPD, despite higher T90 in OVS. In fact, the most striking difference between OVS and OSA alone was in T90. This may indicate less intermittent hypoxia and obstructive events, but on a background of more severe oxygen compromise overall. While this may seem counterintuitive, it may reflect that patients with OVS are vulnerable to impaired gas exchange and its detrimental effects even with fewer upper airway obstructive events compared to similar patients without parenchymal lung disease. In this population T90 may be less of an indicator of OSA severity but more a reflection of the detrimental effects of sleep/lung comorbid pathology. While hypoxic burden is generally a stronger predictor of cardiovascular disease and mortality than AHI or T90 in general OSA patients, T90 may be more relevant in the context of OVS (Azarbarzin et al., 2019). However, further research is needed to determine the relative prognostic value of these metrics for cardiovascular outcomes in OVS.

These differences are likely explained by a relatively milder upper airway collapsibility, less ventilatory response to arousals, and perhaps more responsive upper airway dilator muscles in OVS patients. Conversely, we observed a lower arousal threshold in OVS versus OSA only patients, suggesting it may be a primary driver for OSA in some patients with OVS, but reverse causation (more severe OSA leading to a higher arousal threshold in OSA only patients) is possible as well. Hyperinflation (increased TLC) and air trapping (increased RV and FRC) appeared to be contributors to the protective effect as they were inversely correlated with AHI and/or HB. While increased TLC and RV as well as FRC point toward worsened lung elastance in patients with COPD (Biselli et al., 2015), we suspect that the lung volume changes likely reflect decreased lung elastance and increased lung volume may improve upper airway patency via tracheal traction (Owens et al., 2010). However, for unclear reasons we did not find any significant associations between PFT markers and OSA traits including Vpassive as a marker of upper airway collapsibility. Another possibility is that as lung volume increases there is increased parenchymal destruction and more impaired gas exchange in patients with COPD. This may in turn lead to greater chronic hypoxia and hypercapnia and blunted responses to these influences. While we did not see differences in loop gain between the two groups in our study, a future subset analysis of patients with hyperinflation may be revealing.

Of note, the fact that OSA appeared milder in NREM but not during REM sleep may highlight the importance of accessory muscle use and pharyngeal muscle dilator tone in the OVS population. On the other hand, patients with OVS spent significantly more time during sleep with an oxygen saturation <90% (T90, a marker of both sustained and intermittent hypoxemia), likely due to their underlying lung disorder.

A study of OVS patients led by Orr et al. in 2020 (Orr et al., 2020) evaluated OSA traits using a dedicated physiological research study (CPAP manipulation method) in a matched cohort of 15 patients with OVS and 15 patients with OSA alone. In this smaller study, AHI was also nominally lower (41/h vs. 57/h in OVS vs. OSA only patients, respectively, *p* = 0.17). But no significant differences in OSA traits were found between the two groups, and contrary to our study, there was a trend toward higher arousal threshold and worse upper airway dilator muscle function in OVS patients, as well as several significant associations between PFT markers and OSA traits. These discrepancies may be due to differences in methodology used to estimate traits, sample size, and populations (e.g., higher percentage of women and inclusion of smokers in our study). On the other hand, our findings are mostly consistent with those by Messineo et al. (2020) whose physiological study of 10 OVS patients (CPAP manipulation method) suggested that upper airway collapsibility is generally mild, with arousal threshold being a common driver of OSA pathophysiology. Unlike our current results or those by Orr et al., they also suggested that high loop gain may be a driver in the majority of OVS patients and reported significant associations between arousal threshold and hyperinflation/air trapping; however, the lack of a control group in that study limits potential insights. Overall, these variable findings are likely to reflect that COPD is a heterogeneous disorder encompassing variable degrees of emphysema, small airways disease, skeletal muscle dysfunction, etc. across individuals. Detailed studies connecting COPD endotypes to OSA endotypes may shed additional light on OVS.

Our study has many strengths, notably the sample size and relative diversity suggesting overall good precision and generalizability of our results. While we believe our data to be robust and important, we acknowledge several limitations to our findings. First, several findings suggested substantial effects but did not quite reach statistical significance which suggests that our study may still have been underpowered to detect these differences if such differences truly exist in the underlying populations. Secondly, we recognize that cardiovascular outcomes in patients with OVS are a primary concern for this population. Our study had relatively few cardiovascular indicators. However, we did note a significant increase in mean and maximum pulse rate in patients with OVS which may indicate lower stroke volumes and higher cardiovascular tone during sleep despite lower AHIs and lower sleep apnea‐specific hypoxic burden (but perhaps explained by the higher sustained hypoxemia). Third, we used a method to estimate OSA traits based on routine polysomnography which may be more limited than techniques manipulating upper airway pressure during a research sleep study, but this technique has demonstrated good reliability and provided many important new insights (Alex et al., 2022; Nokes et al., 2024; O'Driscoll et al., 2019; Schmickl et al., 2024). Of note, the sample size of the present study would clearly not be achievable using gold standard invasive physiological methods. Thus, we view our findings as useful and would advocate for further studies using various rigorous methods to encourage further progress in this area. Fourth, our study design was cross‐sectional and thus our findings represent associations. Finally we did not assess the differential effect of positive airway pressure on OVS versus OSA alone, which has been shown to correlate with reduced mortality in OVS (Marin et al., 2010). We encourage further research using experimental manipulations (e.g., NCT05237505) to understand better causal pathways for patients with overlap syndrome and their associated cardiometabolic risk.

In conclusion, OSA severity and mechanisms differ in OVS versus OSA‐only. Patients with more hyperinflation or air trapping have lower AHIs which suggests that more severe COPD related lung changes may be protective with regards to OSA. More research is warranted to assess whether differences in OSA mechanisms affect response to OSA therapy and/or contribute to the worse cardiovascular outcomes.

## FUNDING INFORMATION

Dr. Malhotra is supported by NIH, including R01HL148436. Dr. Schmickl is supported by the American Heart Association (AHA; CDA#940501), the National Institutes of Health (NIH; K23HL161336), and the American Academy of Sleep Medicine Foundation (AASMF; #277‐JF‐22). Dr. Raphelson is supported by the National Institutes of Health (NIH; F32HL176079, NIH Loan Repayment Program Award) and is an ATS ASPIRE fellow. Dr. Marin is supported by Instituto Salud Carlos III, Ministry of Health, Madrid, Spain (PI12/02175, PI15/01940 and PI18/01524). Dr. Sanchez‐Azofra is supported by the Alfonso Martin Escudero Foundation (FUNDAME). Dr. Sands was supported by the NIH (R01HL168067). Dr. Azarbarzin was supported by NIH (R01HL153874) and AASMF (287‐SR‐22). Dr. Orr is supported by the NIH (K23HL151880). The project described was partially supported by the National Institutes of Health, Grant UL1TR000100 of CTSA funding prior to August 13, 2015, and Grant UL1TR001442 of CTSA funding beginning August 13, 2015, and beyond. The content is solely the responsibility of the authors and does not necessarily represent the official views of the AHA, NIH, or AASMF.

## CONFLICT OF INTEREST STATEMENT

Dr. Malhotra is funded by the NIH. He reports income from Eli Lilly, Livanova, Zoll, and Powell Mansfield. He is co‐founder of Healcisio, a small startup in predictive analytics. ResMed gave a philanthropic donation to UCSD. Dr. Orr reports advisory board income from Resmed and Biosency. Dr. Schmickl reports income from consulting for Verily and Apnimed, outside of this work. ResMed provided a philanthropic donation to UCSD. Dr. Owens reports research support from Nitto Denko Asia and Samsung, outside of this work. Dr. Sanchez‐Azofra is currently working for Apnimed, but does not report any conflict of interest. Dr. Raphelson reports no conflict of interest. Dr. Marin reports no conflict of interest. Dr. Sands has worked as a consultant for Apnimed, Respicardia, Forepont, LinguaFlex, Inspire, Eli Lilly, and Nox Medical, and received grant support from Prosomnus, Dynaflex, and Apnimed. He is co‐inventor on intellectual property patented through his institution relating to combination pharmacotherapy (licensed to Apnimed, including royalties) and to sleep apnea phenotyping from wearable technology. His industry interactions are under a management plan by his institution. Dr. Azarbarzin serves as a consultant for Apnimed, Respicardia, Inspire, Eli Lilly, and Cerebra. He is co‐inventor on intellectual property patented through his institution relating to sleep apnea phenotyping from wearable technology. His industry interactions are under a management plan by his institution.

## ETHICS STATEMENT

Data was collected and stored securely, with measures taken to ensure participant confidentiality and anonymity. In cases where participants were from vulnerable populations, specific measures were taken to ensure their rights and well‐being were protected.

## Supporting information


Tables S1–S3.

## Data Availability

The data that support the findings of this study are available from the corresponding author upon reasonable request.
