# Supplementary material for: Obstructive sleep apnea severity and pathophysiological traits in overlap syndrome: Insights from the SNOOzzzE cohort
Source: Physiol Rep. 2025 Jul 3;13(13):e70438. doi: 10.14814/phy2.70438 (PMC12231188; doi:10.14814/phy2.70438)
Supplement: Supplementary file 1 — Tables S1–S3. [file PHY2-13-e70438-s001.docx]

**ONLINE SUPPLEMENT FOR**

**Sleep Apnea Severity and Pathogenesis in OSA-COPD Overlap Patients: a Matched Cohort Study in the SNOOzzzE Cohort**

**Table E1. Fully Adjusted Associations between PFT Measures and OSA Severity Metrics/Traits in OVS Patients who had Complete PFT Data (n=69).** Results are adjusted for age, sex, BMI, race, ethnicity and smoking status. The standardized beta coefficients β_S_ reflect SD-changes in the shown outcome variable for a 1-standard deviation change of the PFT measure.

|  | **FEV1 (%)** | **FVC (%)** | **RV (%)** | **FRC (%)** | **TLC (%)** | **RV/TLC (%)** |
| --- | --- | --- | --- | --- | --- | --- |
| **Outcome Variable** | β_S_  (95%-CI)  P-Value | β_S_  (95%-CI)  P-Value | β_S_  (95%-CI)  P-Value | β_S_  (95%-CI)  P-Value | β_S_  (95%-CI)  P-Value | β_S_  (95%-CI)  P-Value |
| **Primary OSA Severity Metrics** | | | | | | |
| Log(AHI3A), h^-1^ | **0.34** | **0.26** | **-0.28** | **-0.27** | -0.02 | -0.18 |
|  | **(0.1 to 0.58)** | **(0 to 0.52)** | **(-0.52 to -0.04)** | **(-0.53 to -0.02)** | (-0.29 to 0.25) | (-0.42 to 0.07) |
|  | **0.01** | **0.048** | **0.02** | **0.04** | 0.89 | 0.15 |
| Log(HB+1), %min/h | 0.23 | 0.16 | **-0.31** | **-0.3** | -0.14 | -0.2 |
|  | (-0.02 to 0.47) | (-0.1 to 0.42) | **(-0.54 to -0.07)** | **(-0.55 to -0.05)** | (-0.41 to 0.13) | (-0.44 to 0.04) |
|  | 0.07 | 0.22 | **0.01** | **0.02** | 0.3 | 0.1 |
| Log(T90+1), % | -0.2 | -0.21 | -0.04 | -0.04 | -0.21 | 0.01 |
|  | (-0.43 to 0.04) | (-0.46 to 0.03) | (-0.28 to 0.2) | (-0.29 to 0.21) | (-0.46 to 0.04) | (-0.22 to 0.25) |
|  | 0.1 | 0.09 | 0.75 | 0.76 | 0.1 | 0.91 |
| **OSA Traits** |  |  |  |  |  |  |
| Vpassive^T^, %VE | -0.09 | -0.15 | 0.06 | 0 | -0.06 | -0.13 |
|  | (-0.34 to 0.15) | (-0.41 to 0.1) | (-0.19 to 0.3) | (-0.26 to 0.26) | (-0.32 to 0.21) | (-0.37 to 0.11) |
|  | 0.45 | 0.23 | 0.64 | 0.99 | 0.66 | 0.29 |
| Vcomp, %VE | 0.03 | 0.01 | -0.01 | -0.01 | 0.01 | -0.07 |
|  | (-0.22 to 0.27) | (-0.25 to 0.27) | (-0.25 to 0.23) | (-0.27 to 0.24) | (-0.25 to 0.27) | (-0.31 to 0.17) |
|  | 0.81 | 0.94 | 0.95 | 0.92 | 0.95 | 0.55 |
| Loop Gain (dimensionless) | 0.13 | 0.12 | 0.09 | 0.04 | 0.16 | -0.17 |
|  | (-0.12 to 0.37) | (-0.13 to 0.38) | (-0.15 to 0.33) | (-0.22 to 0.3) | (-0.1 to 0.41) | (-0.4 to 0.07) |
|  | 0.3 | 0.33 | 0.45 | 0.76 | 0.23 | 0.16 |
| Arousal Threshold^T^ , %VE | 0.13 | 0.15 | -0.03 | -0.04 | 0.06 | 0.02 |
|  | (-0.11 to 0.37) | (-0.1 to 0.4) | (-0.27 to 0.21) | (-0.3 to 0.22) | (-0.2 to 0.32) | (-0.22 to 0.26) |
|  | 0.3 | 0.24 | 0.82 | 0.75 | 0.64 | 0.88 |
| Ventilatory Response to Arousal, %VE | 0.02 | 0.02 | -0.04 | 0.01 | 0.03 | 0.04 |
|  | (-0.24 to 0.28) | (-0.25 to 0.29) | (-0.3 to 0.22) | (-0.27 to 0.28) | (-0.25 to 0.31) | (-0.22 to 0.29) |
|  | 0.87 | 0.88 | 0.74 | 0.97 | 0.83 | 0.77 |

**Table E2. Results from Univariable and Multivariable Linear Mixed Effects Regression Analyses in Subset of PFT-proven COPD patients who did not use Oxygen during the Overnight Polysomnography (n=256: 64 OVS + 192 matched OSA only).**

| **Outcome Variable** |  | **Univariable Models** | | |  | **Multivariable Models**  (adjusted for race, ethnicity, and smoking) | | |
| --- | --- | --- | --- | --- | --- | --- | --- | --- |
|  |  | **β** | **(95%-CI)** | **P** |  | **β** | **(95%-CI)** | **P** |
| **Primary OSA Severity Metrics** |  |  |  |  |  |  |  |  |
| Log(AHI3A), h^-1^ |  | -0.03 | (-0.23) | 0.73 |  | -0.02 | (-0.22) | 0.86 |
| Log(HB+1), %min/h |  | 0 | (-0.26) | 0.97 |  | 0.07 | (-0.21) | 0.63 |
| Log(T90+1), % |  | **0.49** | **(0.15)** | **0.01** |  | **0.44** | **(0.08)** | **0.02** |
| **OSA Traits** |  |  |  |  |  |  |  |  |
| Vpassive^T^, %VE |  | 2.44 | (-3.78) | 0.44 |  | 1.94 | (-4.63) | 0.57 |
| Vcomp, %VE |  | 3.5 | (-2.31) | 0.24 |  | 3.62 | (-2.43) | 0.25 |
| Loop Gain (dimensionless) |  | -0.01 | (-0.06) | 0.73 |  | -0.02 | (-0.07) | 0.54 |
| Arousal Threshold^T^ , %VE |  | -7.76 | (-16.09) | 0.07 |  | -6.86 | (-15.64) | 0.13 |
| Ventilatory response to arousal, %VE |  | **-10.07** | **(-17.58)** | **0.01** |  | **-9.5** | **(-17.34)** | **0.02** |

**Table E3. Sensitivity Analysis: Bivariable Associations between PFT Measures and OSA Severity Metrics/Traits in OVS Patients who had Complete PFT Data in Subset of PFT-proven COPD patients who did not use Oxygen during the Overnight Polysomnography (n=49).** The standardized beta coefficients β_S_ reflect SD-changes in the outcome variable for a 1-standard deviation change of the PFT measure.

|  | **FEV1 (%)** | **FVC (%)** | **RV (%)** | **FRC (%)** | **TLC (%)** | **RV/TLC (%)** |
| --- | --- | --- | --- | --- | --- | --- |
| **Outcome Variable** | β_S_  (95%-CI)  P-Value | β_S_  (95%-CI)  P-Value | β_S_  (95%-CI)  P-Value | β_S_  (95%-CI)  P-Value | β_S_  (95%-CI)  P-Value | β_S_  (95%-CI)  P-Value |
| **Primary OSA Severity Metrics** | | | | | | |
| Log(AHI3A), h^-1^ | 0.2 | 0.07 | -0.26 | **-0.31** | -0.13 | -0.07 |
|  | (-0.08 to 0.49) | (-0.22 to 0.37) | (-0.54 to 0.02) | **(-0.59 to -0.03)** | (-0.42 to 0.16) | (-0.36 to 0.23) |
|  | 0.16 | 0.62 | 0.07 | **0.03** | 0.37 | 0.65 |
| Log(HB+1), %min/h | 0.05 | -0.05 | -0.27 | **-0.31** | -0.24 | -0.02 |
|  | (-0.24 to 0.35) | (-0.35 to 0.24) | (-0.55 to 0.02) | **(-0.59 to -0.03)** | (-0.52 to 0.05) | (-0.31 to 0.28) |
|  | 0.71 | 0.72 | 0.07 | **0.03** | 0.1 | 0.91 |
| Log(T90+1), % | -0.15 | -0.19 | -0.12 | -0.13 | -0.25 | 0.08 |
|  | (-0.44 to 0.14) | (-0.48 to 0.09) | (-0.41 to 0.17) | (-0.42 to 0.16) | (-0.53 to 0.03) | (-0.21 to 0.37) |
|  | 0.32 | 0.18 | 0.42 | 0.38 | 0.08 | 0.58 |
| **OSA Traits** |  |  |  |  |  |  |
| Vpassive^T^, %VE | -0.08 | -0.08 | 0.11 | 0.07 | 0.05 | -0.13 |
|  | (-0.37 to 0.22) | (-0.38 to 0.21) | (-0.19 to 0.4) | (-0.22 to 0.37) | (-0.24 to 0.34) | (-0.42 to 0.16) |
|  | 0.61 | 0.57 | 0.47 | 0.62 | 0.74 | 0.38 |
| Vcomp, %VE | -0.08 | -0.17 | -0.13 | -0.17 | -0.22 | -0.05 |
|  | (-0.37 to 0.22) | (-0.46 to 0.12) | (-0.42 to 0.16) | (-0.46 to 0.12) | (-0.5 to 0.07) | (-0.34 to 0.25) |
|  | 0.61 | 0.25 | 0.39 | 0.23 | 0.14 | 0.75 |
| Loop Gain (dimensionless) | 0.03 | -0.06 | 0.06 | -0.12 | 0 | -0.08 |
|  | (-0.27 to 0.32) | (-0.35 to 0.23) | (-0.24 to 0.35) | (-0.41 to 0.17) | (-0.29 to 0.29) | (-0.37 to 0.21) |
|  | 0.86 | 0.69 | 0.69 | 0.41 | 0.99 | 0.59 |
| Arousal Threshold^T^ , %VE | 0.05 | 0.01 | -0.1 | -0.17 | -0.12 | 0.04 |
|  | (-0.24 to 0.35) | (-0.29 to 0.3) | (-0.39 to 0.2) | (-0.46 to 0.12) | (-0.41 to 0.17) | (-0.25 to 0.33) |
|  | 0.71 | 0.96 | 0.51 | 0.24 | 0.42 | 0.79 |
| Ventilatory Response to Arousal, %VE | 0.11 | 0.21 | 0.11 | 0.23 | 0.22 | -0.06 |
|  | (-0.19 to 0.4) | (-0.07 to 0.5) | (-0.18 to 0.4) | (-0.06 to 0.51) | (-0.07 to 0.5) | (-0.35 to 0.24) |
|  | 0.47 | 0.14 | 0.45 | 0.12 | 0.14 | 0.7 |
